# Multimodal management of late-stage Bockenheimer disease complicated by severe anemia and coagulopathy: a case report

**DOI:** 10.3389/fmed.2026.1791321

**Published:** 2026-04-30

**Authors:** Zilu Wang, Mingzhe Wen, Qianyun Han, Deming Wang, Lixin Su, Xindong Fan

**Affiliations:** Department of Interventional Therapy, Multidisciplinary Team of Vascular Anomalies, Shanghai Ninth People's Hospital, Shanghai Jiao Tong University, Shanghai, China

**Keywords:** anemia, Bockenheimer disease, case report, coagulopathy, sclerotherapy, venous malformation

## Abstract

**Background:**

Bockenheimer disease is a rare subtype of venous malformations that involves all tissue planes of an upper extremity. This condition progresses throughout life and may cause pain, swelling, hematologic complications, and loss of function. Management for Bockenheimer disease remains challenging.

**Case presentation:**

This study presents a case of a 14-year-old girl with late-stage Bockenheimer disease complicated by severe anemia and coagulation disorder. The patient underwent anticoagulation therapy initially, followed by sclerotherapy combined with local suture ligation twice, and received anticoagulants and molecular targeted medication on maintenance post-discharge. Follow-up after 9 months demonstrated a marked improvement in the general condition and significant volume reduction in the affected limb. Laboratory test results revealed recovery of anemia and well-controlled coagulopathy. Complications included contracture in the left elbow.

**Conclusion:**

Management for Bockenheimer disease requires multimodal therapy, including sclerotherapy, resection, molecular targeted medication, and anticoagulation throughout the entire process. Prompt intervention at an early stage is crucial to the prognosis. Close attention should be paid to the hematologic profiles, especially during the perioperative period.

## 1 Introduction

Venous malformations (VMs) are slow-flow vascular malformations composed of anomalous ectatic veins (1). They are associated with somatic mutations in TEK or PIK3CA (2, 3), leading to defects in the morphogenesis of the venous system. In rare cases where VMs involve all tissue planes of an upper extremity, it is also known as “Bockenheimer disease.” The incidence of VMs is about 1~2:10,000 (4), and the frequency of Bockenheimer disease is unknown.

Bockenheimer disease is a congenital abnormality that progresses throughout life. Usually, asymptomatic blueish masses were first noted in early childhood, which progressively enlarged as the child grew. The engorged, bulky limb might cause disfiguration, swelling, pain, limited joint mobility, as well as complications including consumptive coagulopathy and pathological fracture (5).

Management for Bockenheimer disease remains challenging. Although various approaches, including compression, sclerotherapy, resection, and medication, have been proven effective, these treatments are merely palliative and cannot cure this complex condition (5). Moreover, given a secondary systematic coagulopathy, treatment itself might cause severe thrombohemorrhagic complications, such as massive hemorrhage, thromboembolism, or even disseminated intravascular coagulation (6).

Here, we present a complex case of late-stage Bockenheimer disease in a 14-year-old girl, who showed a significant and inspiring outcome following multimodal therapy. This study aims to characterize an appropriate multimodal management of Bockenheimer disease, and underlines the necessity of prompt intervention at an early stage in this patient group.

## 2 Case presentation

### 2.1 Clinical presentation and evaluation

**Figure 1:**
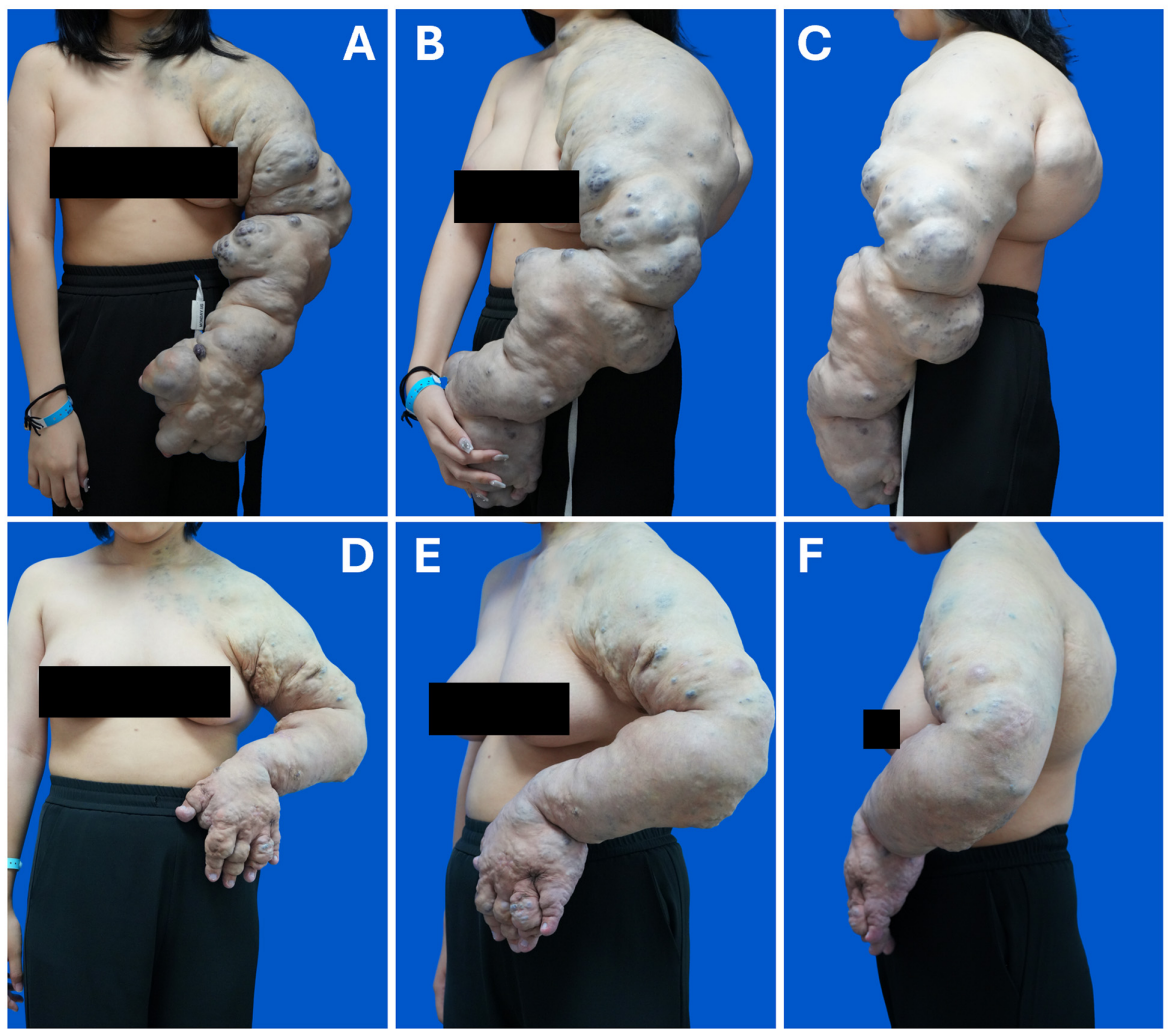
Extensive venous malformations of the left upper extremity in a 14-year-old girl. Front **(A)**, oblique **(B)**, and side **(C)** portraits at first visit. The extensive blueish lesions involved the entire left upper extremity and extended to the left neck and thoracic wall. Front **(D)**, oblique **(E)**, and side **(F)** portraits at 9-month follow-up. The bulky extremity exhibited a significant reduction. Contracture developed in the left elbow joint.

A 14-year-old girl was referred to our clinic with extensive blueish lesions involving the left upper extremity. The lesions were noted at birth and had progressively enlarged since, causing functional impairment of the extremity without pain. On examination, the patient was profoundly weak and unable to tolerate even brief periods of standing. Marked pallor was noted, particularly in the conjunctivae and lips. The engorgement of the left upper extremity was soft and compressible without tenderness or pulsation, extending to the left shoulder, neck, and chest wall (Figures 1A–C). Active movement was nearly absent in all directions at the left shoulder, elbow, and wrist joints, with complete functional loss of the left hand.

**Figure 2:**
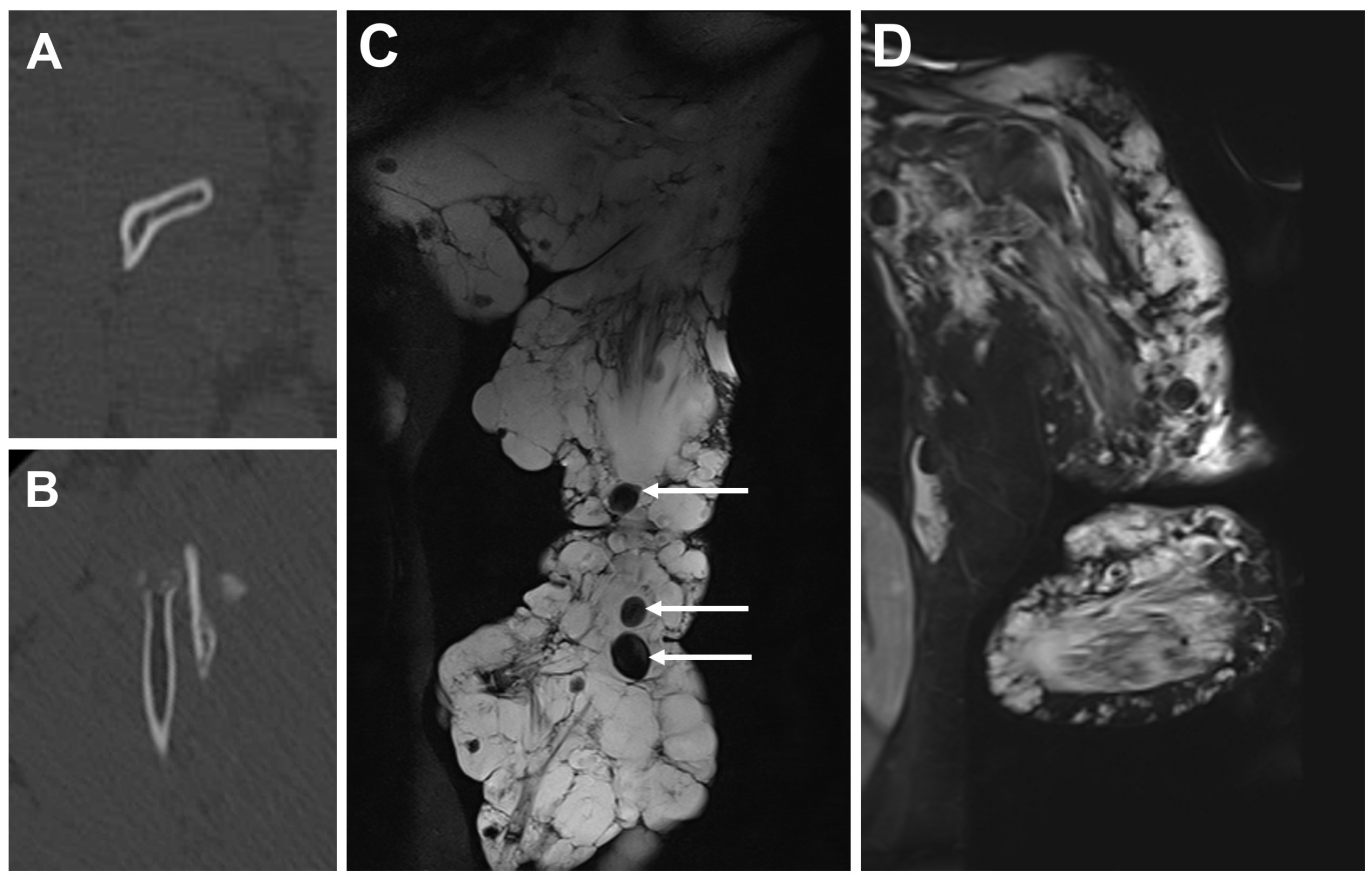
Radiology findings on admission. Computed tomography with bone window settings demonstrated radiolucency in the medulla of the left humerus **(A)** and radius **(B)**. T2-weighted magnetic resonance imaging **(C)** revealed a multicystic, marked hyperintensity infiltrating the left upper extremity. Phleboliths presented as signal voids (arrows). Magnetic resonance imaging at 9-month follow-up **(D)** showed a decrease in signal intensity compared to **(C)**.

Computed tomography (CT) showed sparse trabeculae in the bones of the left upper limb, while cortex integrity remained preserved (Figures 2A, B). Magnetic resonance imaging (MRI) revealed abnormal, dilated veins in the left upper extremity, with multiple phleboliths interspersed throughout (Figure 2C). Laboratory examination revealed a significant coagulation disorder, with prolonged coagulation times, a D-dimer level of 51.10 mg/L fibrinogen equivalent unit (reference value < 0.5), unmeasurable fibrinogen levels (reference value 1.8–3.5 g/L), yet only mild thrombocytopenia (100 × 10^9^/L, reference value 125–350 × 10^9^/L). The blood routine test revealed a severe anemia, with a red blood cell (RBC) count of 3.21 × 10^12^/L (reference value 3.80–5.10 × 10^12^/L) and a hemoglobin level of 62 g/L (reference value 115–150 g/L). Detailed laboratory test results on admission are shown in Table 1.

**Table 1:** Laboratory test findings on admission.

Test	Result	Reference value
RBC ( × 10^12^/L)	3.21	3.80–5.10
Hemoglobin (g/L)	62	115–150
WBC (10^9^/L)	2.52	3.50–9.50
Platelet (10^9^/L)	100	125–350
MCV (fL)	71	82–100
MCH (pg)	19.3	27–34
MCHC (g/L)	272	316–354
Reticulocyte count ( × 10^12^/L)	0.14	0.021–0.098
Peripheral blood smear		
Anisocytosis	1+	
Hypochromia	2+	
Schistocytes	2+	
PT (s)	Unmeasurable^*^	9–13
APTT (s)	46.2	20–40
TT (s)	83.7	14–21
D-dimer (mg/L FEU)	51.10	< 0.5
Fibrinogen (g/L)	Unmeasurable^*^	1.8–3.5
TB (μmol/L)	36.0	0–21
DB (μmol/L)	6.8	0–4
IB (μmol/L)	29.2	0–17
ALT (U/L)	4	6–29
AST (U/L)	8	10–31
Lactate dehydrogenase (U/L)	187	100–240
Haptoglobin (g/L)	0.48	0.30–2.00
Urobilinogen	1+	negative
Antiglobulin test	negative	negative

RBC, red blood cell; WBC, white blood cell; MCV, mean corpuscular volume; MCH, mean corpuscular hemoglobin; MCHC, mean corpuscular hemoglobin concentration; PT, prothrombin time; APTT, activated partial thromboplastin time; TT, thrombin time; FEU, fibrinogen equivalent unit; TB, total bilirubin; DB, direct bilirubin; IB, indirect bilirubin; ALT, alanine aminotransferase; AST, aspartate aminotransferase.

^*^Unmeasurable due to the absence of clot formation.

### 2.2 Diagnosis and treatment plan

The patient was diagnosed with diffuse VMs of the left upper extremity (Bockenheimer disease), anemia, and coagulopathy. The abnormalities of the hematological system were probably secondary to the massive VMs. During the consultation, the patient and her guardians expressed a strong willingness to attempt limb salvage. Sequential therapy was scheduled as follows after interdisciplinary meetings and fully informed consent: (1) correction of coagulopathy and anemia; (2) stepwise sclerotherapy combined with local suture ligation; (3) long-term molecular targeted medication. Compressive garments were also attempted but not pursued, as the patient experienced intolerable pain when pressure was applied.

### 2.3 Multimodal management

The severe consumptive coagulopathy at baseline posed risks of spontaneous hemorrhage and thromboembolism, meanwhile significantly increased treatment risks. Thus, anticoagulation with enoxaparin (6,000 IU/0.6 mL subcutaneous injection, twice daily) was initiated. However, the patient's coagulopathy remained refractory, with a fibrinogen level of 0.58 g/L (reference value 1.8–3.5 g/L) after 2 weeks of continuous anticoagulation. Consequently, intravenous human fibrinogen (2 g daily) was added to the treatment regimen, followed by a transfusion of 5 units of cryoprecipitate. Simultaneously, anemia was also corrected by transfusion. After transfusion of 1 unit of packed RBCs on post-admission days 2, 4, and 11, hemoglobin levels improved to 84 g/L.

After fibrinogen stabilized above 1 g/L, the patient underwent two sessions of sclerotherapy combined with local suture ligation, with an interval of 1 month. All procedures were performed under general anesthesia with a standard aseptic technique. Local suture ligations were performed at the most prominent sites using 3-0 absorbable sutures (Supplementary Figure 1, Supplementary Video 1). Butterfly needles were accessed adjacent to the ligation sites via direct percutaneous puncture. The specific regimens for the two sclerotherapy sessions were as follows: 2 mL of polidocanol for the first session, and 15 mL of absolute ethanol for the second session (Supplementary Table).

### 2.4 Complications

**Figure 3:**
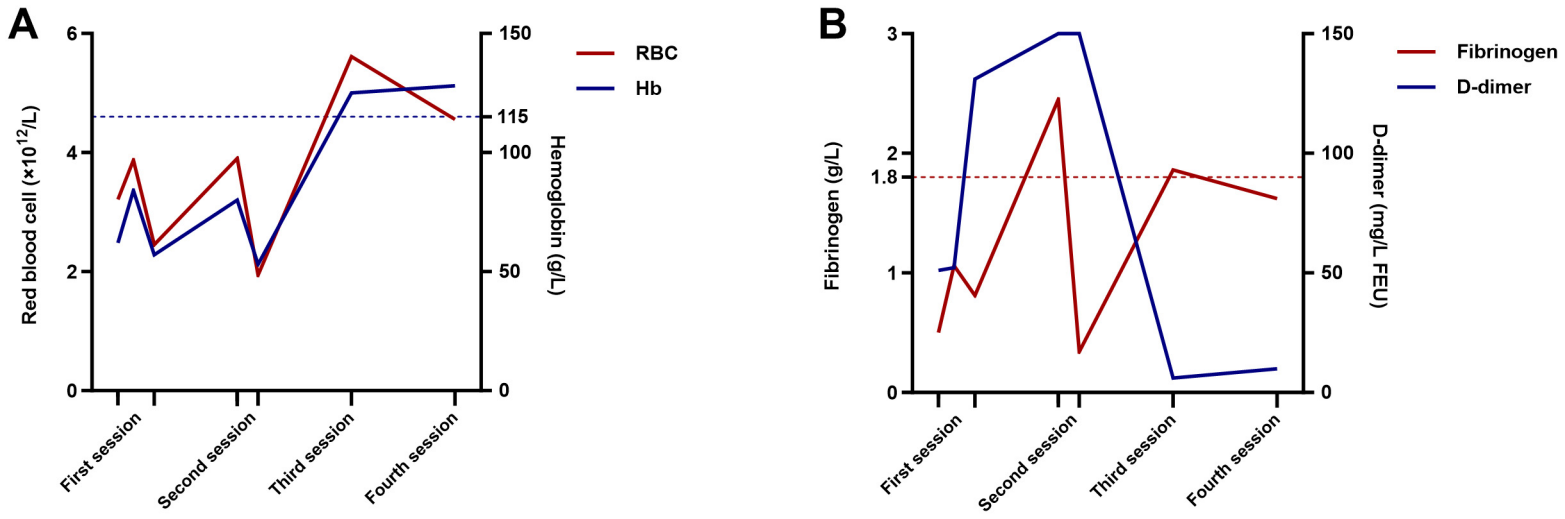
Trends of laboratory indicators related to anemia and coagulopathy. Laboratory investigation revealed recovery of anemia and well-controlled coagulopathy following the first two treatment sessions. **(A)** Levels of red blood cells and hemoglobin declined abruptly after the first and second sessions, and reached the reference range before the third session. **(B)** Trends of fibrinogen and D-dimer. Fibrinogen level peaked before the second session, probably owing to the transfusion of human fibrinogen, and decreased drastically after treatment. Afterwards, it improved significantly during the interval, and touched the lower limit of the reference value before the third session. D-dimer levels increased dramatically following the first treatment and exceeded the detection limit during the second session. Similarly, it showed great improvement during the interval, but was still markedly elevated before the third session. RBC, red blood cell; Hb, hemoglobin; FEU, fibrinogen equivalent unit.

The following postoperative symptoms arose during the interval between the two treatment sessions. Swelling, a common postoperative symptom of sclerotherapy, was noted in her left upper limb as expected. Pain and fever were managed by naproxen sodium (0.275 g intravenous drip, once daily). Laboratory investigations on the first postoperative day revealed an abrupt decrease of hemoglobin (57 g/L) and fibrinogen (0.81 g/L) (Figure 3). Therefore, Transfusions of 1 unit of packed RBCs, 200 mL of fresh frozen plasma, and 5 units of cryoprecipitate were administered. Moreover, the patient developed generalized jaundice 2 days after the first treatment session. Despite a significant elevation in bilirubin (direct bilirubin 14.8 μmol/L, reference value < 4 μmol/L; indirect bilirubin 82.6 μmol/L, reference value < 17 μmol/L), the alanine aminotransferase and aspartate aminotransferase remained within the normal range. Combined with the abdominal ultrasound findings and a history of sclerotherapy, an acute hemolysis might explain the mechanism of jaundice. The manifestation subsided 1 week after the procedure with the aid of ademetionine (1,000 mg intravenous drip, once daily). Part of the ligation sites developed ulceration, which underwent careful debridement during the second treatment session.

Postoperative symptoms had substantially resolved by the time of discharge. The patient was prescribed rivaroxaban (15 mg daily) and sirolimus (2 mg daily) for maintenance post-discharge. Blood concentration monitoring of sirolimus showed an effective concentration above 8 ng/mL, with no obvious side effects.

### 2.5 Follow-up results and retreatment

At the 9-month follow-up, the patient exhibited marked improvement in general condition and psychological state, progressing from wheelchair dependency to toleration of light physical activity. The lesion volume in the left upper extremity showed a significant reduction (Figures 1D–F, 2D); however, severe contracture developed in the left elbow joint. Blood routine and coagulation tests revealed normal results, except for the D-dimer level (6.10 mg/L fibrinogen equivalent unit, reference value < 0.5) and fibrinogen degradation products (17.65 μg/mL, reference value < 5 μg/mL) (Figure 3). Another two sessions of sclerotherapy were scheduled under general anesthesia. Phleboliths that were superficial to the skin were resected. The patient remains under follow-up care.

Protocols and complications of each treatment were listed in the Supplementary Table. The treatment timeline is demonstrated in Supplementary Figure 2.

## 3 Discussion

In 1907, a 52-year-old male with progressive prominence of the veins of the left upper limb was reported by a German surgeon, Philipp Bockenheimer. The patient underwent resection, followed by amputation 3 months later, but eventually died likely due to infection and bleeding. Bockenheimer described this pathological condition as “genuine (congenital) diffuse phlebectasia” after dissection, which was termed “Bockenheimer's disease” later by his colleague and has remained in use since then (7).

Bockenheimer disease is a rare subtype of VMs characterized by involvement of all tissue planes of an extremity, including the skeletal system. Phlebectasia was prominent in the affected limb, while the arterial system remains in a physiological status (5). Like VMs, Bockenheimer disease presents as bluish, compressive masses that engorge with a dependency on position. This condition progresses throughout life and may cause pain, swelling, joint stiffness, and even loss of function (5). This study presents a girl with Bockenheimer disease who didn't receive prompt and appropriate intervention at an earlier age, thus unfortunately progressing into an advanced stage with severe hematologic complications. At the initial visit, our patient complained of chronic, persistent swelling without spontaneous pain. The function of her left upper limb had been nearly completely lost owing to the mass effect.

Almost all the patients of Bockenheimer disease are complicated by a specific type of coagulopathy, termed localized intravascular coagulation (LIC). Blood stagnation inside the lesion leads to a constant activation of the coagulation system, therefore the fibrinolysis system, reflected by hypofibrinogenemia and an elevated D-dimer level in the laboratory tests (8). However, the platelet count could be normal or merely slightly decreased. The laboratory findings of our patient at baseline typically reflected the characteristic features of LIC (Table 1). Notably, LIC associated with VMs should be differentiated from the Kasabach-Merrit phenomenon in tufted angioma and Kaposiform hemangioendothelioma (9). These are vascular tumors in infants, characterized by profound thrombocytopenia (10). Another manifestation of LIC is the phleboliths inside VMs lesions, which resulted from calcification of thrombus (11). Phleboliths appear as high-density foci on radiographs and CT scans, and signal voids on MRI (Figure 2C).

Though infrequent, LIC could progress into massive hemorrhage, deep venous thrombosis, or even disseminated intravascular coagulation under the circumstances of treatment (12). Therefore, screening coagulation profiles and correcting LIC preoperatively are essential. In our clinical practice, intervention is not considered until the fibrinogen level exceeds 1 g/L. Low-molecular-weight heparin is the first-line therapy for LIC (13). The standard therapeutic protocol for LMWH in managing LIC is subcutaneous injection at a weight-based dose of 100 IU/kg twice daily. In this case, considering the patient's adolescent age and the high risk of spontaneous hemorrhage, a slightly conservative dose of 6,000 IU (approximately 90 IU/kg) twice daily was initiated. Despite this continuous anticoagulation, the patient's coagulopathy remained refractory after 2 weeks. Supportive treatment of transfusion is a direct and effective treatment of LIC. Fresh frozen plasma and cryoprecipitate could replenish the coagulation factors that are constantly being consumed. Comparatively, liquid plasma shows a limited effect due to the degradation of factor V, VIII. Recent studies have indicated that direct oral anticoagulants showed promising treatment effects in the management of LIC, including rivaroxaban, dabigatran etexilate, etc. They might serve as an alternative to LMWH for patients requiring long-term maintenance therapy, but high-quality evidence from randomized controlled trials regarding their efficacy and safety is still lacking. In this study, the patient was prescribed rivaroxaban post-discharge. In conclusion, given a severe and intractable coagulopathy on admission, the patient underwent the perioperative period without significant thrombohemorrhagic complications, and showed great improvement in coagulation profiles at the 9-month follow-up (Figure 3).

Anemia is rarely seen in VMs and may be under-recognized as a complication of Bockenheimer disease. Blood routine results on admission revealed severe anemia, with a hemoglobin level of 62 g/L. Combined with other laboratory findings (Table 1), we deemed that pathological conditions of hemorrhage and hemolysis co-existed in the affected limb and jointly contributed to the anemia. Microcytic hypochromic anemia (MCV 71 fL, MCH 19.3 pg) indicated the presence of chronic bleeding, which was morphologically confirmed by hypochromia (2+) on the peripheral blood smear. Simultaneously, though lactate dehydrogenase (187 U/L) and haptoglobin (0.48 g/L) remained within the reference range, elevated reticulocyte count (0.14 × 10^12^/L), indirect bilirubin level (29.2 μmol/L), and the presence of urobilinogen (1+) all indicated the presence of compensated hemolysis. Furthermore, schistocytes (2+) on the blood smear strongly suggested a mechanical hemolytic process within the malformed veins. As mentioned above, blood stagnation within the ectasia veins activated the coagulation system that brought about thrombosis. Shear stress from fibrin thrombus caused RBC fragmentation, leading to a hemolysis process which resembled the pathogenesis of microangiopathic hemolytic anemia (14). Meanwhile, the excessive consumption of coagulation factors and fibrinogen led to intralesional occult bleeding. In the presented case, hemoglobin levels improved to 84 g/L (reference value 115–150 g/L) after transfusions of packed RBCs preoperatively, and stabilized above 100 g/L following two sessions.

Resection of lesions, or amputation, posed significant difficulties and risks in this case. The malformed lesion extended to the left shoulder, neck, and chest wall, leading to a dilemma when choosing an ideal amputation level. Besides, there were high risks of both intraoperative hemorrhage and unsuccessful wound healing. Coupled with the patient's strong willingness for limb salvage, we eventually opted for multi-session sclerotherapy. This approach represents the first-line therapy of VMs due to its established efficacy and minimally invasive nature (15). Localization of aberrant veins was straightforward in this patient; thus, venography was only performed in the last two sessions. In consideration of treatment safety, the dosage of sclerosant was gradually increased across the four sessions. Absolute ethanol possesses the most potent efficacy among all the sclerosants; thus it was chosen in the second, third, and fourth sessions. During the initial two sessions, local suture ligation was performed to enhance the effectiveness of sclerotherapy. Sutures created artificial septa within the venous sac, thereby prolonging the reaction time of the sclerosants. Similar techniques have been reported to improve thrombocytopenia in the management of Kaposiform hemangioendothelioma (16, 17).

Sirolimus, an inhibitor of mammalian target of rapamycin (mTOR), has been demonstrated to be safe and efficacious in patients with slow-flow vascular malformations (18, 19). This effect is dependent on the molecular basis of an aberrant activation of the phosphatidylinositol 3-kinase (PI3K)/protein kinase B (AKT)/mTOR pathway (20). At present, there is an absence of clinical trials regarding the application of sirolimus targeting patients with extensive VMs. However, evidence supported the efficacy of sirolimus in improving coagulation parameters in patients with slow-flow vascular malformations of heterogeneous diagnosis (21). Although patient-specific genetic data were not available, the decision to initiate sirolimus was made based on our current genetic knowledge of Bockenheimer disease (22), aiming to slow the progression of VMs and improve her coagulation profile.

Postoperative symptoms and complications were summarized in the Supplementary Table. Postoperative symptoms included pain, swelling, jaundice, ulcer, etc. Jaundice was caused by an acute hemolysis following sclerotherapy and subsided after 1 week. Ulceration developed in some of the ligation sites and was treated with topical antibiotics and debridement. Transient consumption of hemoglobin and coagulation factors was managed with transfusions. Overall, postoperative symptoms were generally manageable and gradually diminished over the course of treatment. Unfortunately, contracture in the left elbow developed after the first two treatment sessions (Figures 1D–F), which could be attributed to a non-selective fibrotic effect of the sclerosants on adjacent tendons. Notably, the extensive involvement of multiple tissue planes, including the entire joint, leads to a predisposition to joint contracture in Bockenheimer disease, whether through natural disease progression or secondary to interventions such as sclerotherapy and surgical resection (23, 24). Literature has reported an incidence of contracture as high as 61.9% (13/21) following surgical intervention in this patient population (5). While post-procedural physical therapy or splinting are recommended to alleviate such symptoms (23), expectant treatment was adopted in the presented case considering a high risk of pathological fracture.

In summary, the patient underwent a multimodal therapy containing anticoagulation, local suture ligation, sclerotherapy, and molecular targeted medication (Supplementary Figure 2). At the 9-month follow-up, she exhibited marked improvement in both lesion volume and hematologic profiles. However, several limitations of this report warrant consideration. First, standardized quantitative measures such as serial limb volumetry or quality-of-life questionnaires were lacking, which could have provided a more objective evaluation of the treatment efficacy. Second, while the diagnosis of iron-deficiency anemia was supported by erythrocyte indices and blood smear morphology, definitive biochemical confirmation through serum ferritin and iron measures was not available. Finally, due to the lack of willingness from the patient and her guardians to undergo genetic testing, we were unable to obtain the genetic information that could guide her pharmacotherapy. It is also important to acknowledge that the significant clinical improvement and stabilization of the patient's coagulopathy cannot be attributed to any single intervention. Rather, the successful outcome likely reflects the synergistic effect of a multimodal therapeutic strategy. This highlights the necessity of a comprehensive, multidisciplinary approach in managing late-stage Bockenheimer disease.

## 4 Conclusion

This study presents a case of extensive VMs (Bockenheimer disease) in its advanced stage, which showed a significant and inspiring outcome following multimodal therapy. Management for Bockenheimer disease includes anticoagulation, compression, sclerotherapy, resection, molecular targeted medication (sirolimus, alpelisib), etc. Although Bockenheimer disease cannot currently be cured, prompt intervention at an early age could significantly slow the progression. Close attention should be paid to the hematologic profiles, especially during the perioperative period.

## Data Availability

The original contributions presented in the study are included in the article/Supplementary material, further inquiries can be directed to the corresponding author.
