# Supplementary figures and images for: Multimodal management of late-stage Bockenheimer disease complicated by severe anemia and coagulopathy: a case report
Source: Front Med (Lausanne). 2026 Apr 30;13:1791321. doi: 10.3389/fmed.2026.1791321 (PMC13171330; doi:10.3389/fmed.2026.1791321)

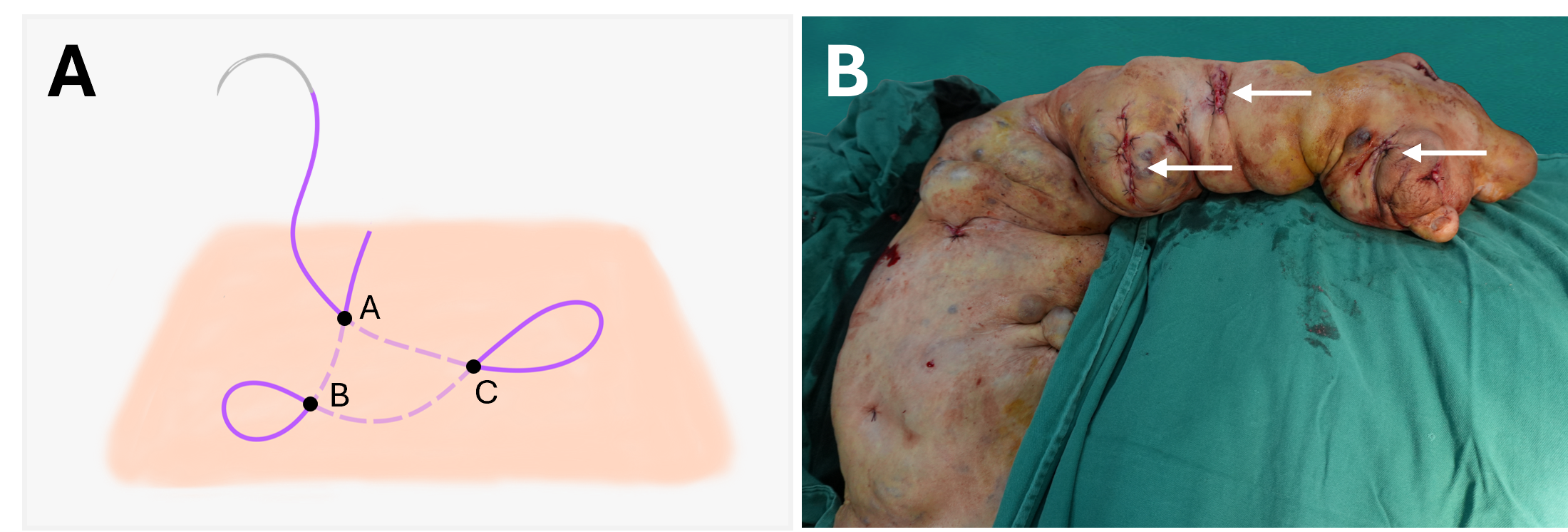

Supplement: Supplementary file 1 [file Image_1.tif]

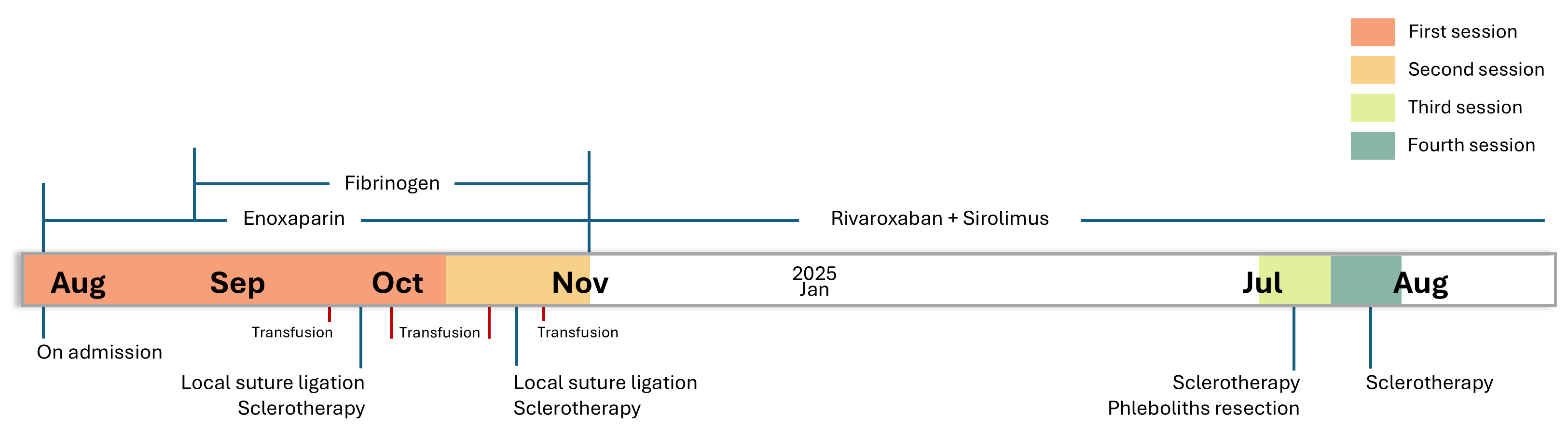

Supplement: Supplementary file 2 [file Image_2.tif]
