# Supplementary material for: Multimodal management of late-stage Bockenheimer disease complicated by severe anemia and coagulopathy: a case report
Source: Front Med (Lausanne). 2026 Apr 30;13:1791321. doi: 10.3389/fmed.2026.1791321 (PMC13171330; doi:10.3389/fmed.2026.1791321)
Supplement: Supplementary file 3 [file Table_1.docx]

**Supplementary Table**

Treatment protocols, post-operative symptoms and complications

| **Date** | **Treatment protocols** | **Post-operative symptoms** | **Post-operative complications** |
| --- | --- | --- | --- |
| 2024.9 | Local suture ligation,  sclerotherapy (2 mL of polidocanol mixed with air to generate 10 mL of foam) | Pain, fever, swelling | Jaundice, ulcer, decrease in hemoglobin and fibrinogen |
| 2024.10 | Local suture ligation, debridement,  sclerotherapy (15 mL of absolute ethanol) | Pain, fever, swelling | Ulcer, decrease in hemoglobin and fibrinogen, elbow contraction (long term) |
| 2025.7 | Sclerotherapy (4 mL of polidocanol, 8 mg of bleomycin, 30 mL of absolute ethanol),  phleboliths resection | Pain, swelling | None |
| 2025.8 | Sclerotherapy (4 mL of polidocanol, 8 mg of bleomycin, 30 mL of absolute ethanol) | Pain, swelling | None |
